# Screening for alveolar echinococcosis in household members of affected patients: A cross-sectional study with a pooled comparison to endemic population screenings

**DOI:** 10.1016/j.onehlt.2026.101544

**Published:** 2026-08-16

**Authors:** Lynn Peters, Alexander Grunenberg, Giuseppe Zenatti, Wanjie Jiang, Yasemin Özkan, Nele Wellinghausen, Teresa Rangel-Vivar, Andreas Binzberger, Wolfgang Kratzer, Peter Kern, Benedikt Haggenmüller, Beate Grüner

**Affiliations:** a University Hospital Ulm, Department of Internal Medicine III, Division of Infectious Diseases, Albert-Einstein-Allee 23, 89081 Ulm, Germany; b MVZ Labor Ravensburg GbR Labor Dr. Gärtner, Elisabethenstraße 11, 88212 Ravensburg, Germany; c University Hospital Ulm, Department of Internal Medicine I, Albert-Einstein-Allee 23, 89081 Ulm, Germany; d University Hospital Ulm, Department of Radiology, Albert-Einstein-Allee 23, 89081 Ulm, Germany

**Keywords:** Alveolar echinococcosis, Echinococcus multilocularis, Household contacts, Screening, Seroprevalence

## Abstract

**Background:**

Alveolar echinococcosis (AE) is an emerging and potentially fatal zoonosis caused by accidental ingestion of infective eggs of the cestode *Echinococcus multilocularis.* Household members of AE patients share the same human–animal–environment interface and may therefore have similar risks for exposure to infective eggs.

**Methods:**

We conducted a cross-sectional study at the University Hospital of Ulm, Germany. Unrelated household members of AE patients who shared the same household for ≥5 years prior to the patient's diagnosis were invited for screening with serological testing and abdominal ultrasound. The primary endpoint was the prevalence of possible or probable AE.

**Results:**

A total of 100 household members from 100 AE patients were recruited. Six participants showed positive or borderline results in one of the two serological tests, but none showed positivity in both and all had negative western blot results. Of the 88 participants with available imaging, only one participant had a calcified, inactive AE lesion, classified as possible AE. No participant met the criteria for probable AE.

**Conclusion:**

We observed a higher seroprevalence (6.0%) among household members than pooled estimates from population-based screenings in high-endemic areas (1.9%; 95% CI 1.6–2.1%). The prevalence of inactive AE (1136 per 100,000) also exceeded that reported in these studies (51 per 100,000; 95% CI 22–101); however, the sample size and a different screening approach limit the comparability of the findings. Importantly, AE remained rare in this group, and no clinically relevant active cases were detected. Although exposure to infective EM eggs may be increased among household members, host-related factors may influence whether infection becomes clinically manifest. Based on these results, routine screening of unrelated household members cannot be recommended at this time.

## 1 Background

### 1.1 *E. multilocularis* transmission and human exposure risk factors

Alveolar echinococcosis (AE) is a rare, but emerging zoonotic disease caused by the cestode *Echinococcus multilocularis* (EM). The adult parasite resides in the intestinal tract of red foxes (*Vulpes vulpes*) and releases infective eggs into the environment with the fox's faeces, while rodents serve as natural intermediate hosts. Through accidental ingestion of these eggs by contaminated hands or food, humans can serve as accidental intermediate hosts, developing the metacestode (larval stage) with invasive tumor-like growth primarily in the liver [1]. Environmental risk factors for human exposure to EM eggs include dog or cat ownership, residence on a farm or in proximity of agricultural fields, agricultural or forestry work, kitchen gardening, among others [2], [3], [4]. This transmission cycle can be understood within a One Health framework, as human infection depends on interactions between wildlife hosts, domestic animals, environmental contamination, and human behaviour.

Household members of AE patients usually share their living and sometimes working conditions. Sharing a household may therefore serve as a proxy for common environmental and animal-related exposures, including use of the same garden or agricultural surroundings, contact with the same domestic animals, and exposure to environments contaminated with EM eggs. Household members of AE patients may thus be considered a potential high-risk population for EM infection.

### 1.2 Clinical observations and study objective

At our specialized AE centre, we observed two cases in which unrelated household members of patients with AE were also diagnosed with the disease. In the first case, the spouse of an AE patient underwent serological testing and abdominal ultrasound in 2007 because of potential exposure associated with regular hunting activities and dog ownership. Both examinations were negative. Eight years later, routine laboratory testing revealed elevated gamma-glutamyltransferase levels, prompting abdominal ultrasound, which identified several active hepatic AE lesions measuring up to 60 mm. The second case concerned a couple living in a rural area near a forest with regular fox sightings who owned cats and had a kitchen garden. After AE had been diagnosed incidentally in one partner, the other partner underwent abdominal ultrasound at the recommendation of the family physician, revealing an active hepatic AE lesion.

These observations prompted the question of whether screening for AE in household members should be recommended. AE typically remains asymptomatic for five to ten years [5], and early detection through screening of asymptomatic at-risk individuals could enable timely treatment, halt disease progression, allow for surgical resection, and improve outcomes for AE patients. At symptom onset, the disease is often advanced, limiting treatment options and increasing the risk of complications, which adversely affects quality of life and increases morbidity and mortality [6], [7], [8], [9].

To the best of our knowledge, no study has yet investigated the prevalence of AE in household members with similar environmental risk factors, and no guideline has addressed the possible benefit of screening in this presumed high-risk population.

### 1.3 Screening modalities and diagnostic implications

Screening can be performed by serological testing or US, or a combination of both. The sensitivity of conventional US is estimated to be rather high with 95% (90.7%–99.3%), yet the specificity rather low at 20.7% (6.0%–35.4%) [10] since it needs an experienced examiner [11]. Typical findings include hyper- and hypoechogenic areas in a pseudo-tumor with irregular limits and scattered calcification, pseudo-cystic appearances due to a large area of central necrosis, hemangioma-like or metastasis-like lesions or a small calcified lesion, which is considered an inactive aborted lesion [11], [12]. The specificity can be increased using contrast-enhanced US (CEUS) to 81.3% when comparing AE with cholangiocellular carcinoma [13] and up to 100% when comparing to heamangioma [14] or metastases [15], which are all common differential diagnoses for AE.

As for serological tests, the use of recombinant EM antigens has a high diagnostic sensitivity of 90 to 100%, with a specificity of 95 to 100%. Immunoblots containing lysate or recombinant antigens can be used as additional confirmation tests to increase specificity [16]. To establish the diagnosis, the combination of a high-sensitivity serological screening test and a high-specificity serological test is recommended [11].

A lesion typical for AE along with two positive serological tests allows the diagnosis of a probable AE and requires treatment. If only the serological tests turn out positive, the patient is classified as possible AE. Positive serological tests without corresponding parasitic lesion can indicate undergone infection with EM without establishment of the disease (yet). These cases do not qualify for anthelminthic treatment, however, should be monitored in case an active disease develops. A single positive serological test is usually referred to as seropositivity, but may be nonspecific and does not allow a diagnosis of possible AE. If US reveals an early or inactive calcified lesion, the serological tests can remain negative, which also is labeled as possible AE, leading to a ‘watch and wait’ strategy [11].

## 2 Methods

### 2.1 Study design

We conducted a cross-sectional screening study at the University Hospital of Ulm, Germany, between 04/2024 and 05/2025. All AE patients coming to the out-patient department received an invitation to participate in the study prior to their scheduled visit. In case one of their household members met the inclusion criteria, they joined the AE patients for the next visit and were offered serological testing and an abdominal US. If an abdominal US, computed tomography (CT), or magnetic resonance imaging (MRI) scans was available from up to 6 months prior to study inclusion, these findings were taken into account and the US was not repeated.

Additionally, we describe the characteristics of two pairs of household members known to have AE from the AE cohort at our centre, who were diagnosed prior to this screening.

The study has been approved by the ethics committee of the University of Ulm (no. 20/24) and participants were included after obtaining written informed consent.

### 2.2 Inclusion criteria

Inclusion criteria for household members were 1.) age ≥ 18 years, 2.) no genetic relationship to the AE patient, and 3.) sharing a household with the respective AE patient for ≥5 years prior to the AE diagnosis. The second criterion aimed at excluding genetic influences and the third criterion ensured that household members shared the same environmental exposures during the presumed timeframe when the AE patient acquired the infection [5].

### 2.3 Study endpoint

The primary endpoint of the study was the prevalence of non-related household members with possible or probable AE. Possible AE is defined as positive specific serology for AE in two different serological tests OR an abdominal US typical for AE. To meet the case definition ‘probable’ both the serological tests AND the abdominal imaging must suggest AE [11].

### 2.4 Screening methods

For serological testing, we used the *Echinococcus* IgG EIA (Virion/Serion ELISA classic *Echinococcus* IgG ESR107G, Würzburg, Germany), and the Em2-Em18 IgG ELISA (Bordier Affinity, Crissier, Switzerland). The former contains native hydatid cyst antigen from *E. granulosus*, and the latter uses purified extract of in vitro cultured vesicles from EM (Em2) in combination with recombinant Em18 antigen. Manufacturer-reported sensitivities and specificities are 99% and 97.1% for the *Echinococcus* IgG ELISA and 83% and 98% for the Em2-Em18 ELISA, respectively. As a third method, we used a commercial western blot in order to clarify positive or borderline cases in the ELISA tests. The *Echinococcus* western blot (LDBio Diagnostics, Lyon, France) contains EM larval antigen extract and detects IgG antibodies against *E. granulosus* and *EM*, also allowing its differentiation. Manufacturer-reported sensitivities and specificities are 97.3% and 94.6%.

Abdominal US was performed using convex transducers (C5–1 MHz, C9–1 MHz) on the following systems: Canon/Toshiba Aplio 500 and Aplio i800 (Canon Medical Systems Corporation, Otawara, Japan), Hitachi HI VISION Ascendus (Hitachi Medical Systems Americas, Twinsburg, Ohio, USA), Philips EPIQ 7G (Philips Medical Solutions, Mountain View, California, USA), Siemens Acuson Sequoia (Siemens Healthineers, Erlangen, Germany), and Samsung RS85 Prestige (Samsung Medison, Seoul, South Korea). Image data were documented using the ViewPoint Ultrasound (VPU) reporting system (GE Healthcare, Munich, Germany). All examinations were conducted in our interdisciplinary ultrasound centre, jointly operated by the Clinics of Radiology and Internal Medicine. In addition to residents in training, three senior sonographers – each with many years of experience, especially in imaging of echinococcosis – were available to all examiners for consultation and supervision during image acquisition and interpretation. In cases of inconclusive liver findings, CEUS was performed to further characterize the lesions.

### 2.5 Statistical analysis

Demographic results are presented with mean, median, standard deviation (SD) and range and were calculated with IBM SPSS V25. The Clopper-Pearson 95% confidence intervals (95% CI) were estimated using Epitools Epidemiological Calculators, Ausvet, Sergeant, ESG, 2018. All other calculations were performed with Microsoft Excel 365. Analyses were conducted using pairwise deletion for missing data.

## 3 Results

After inviting the household members of 387 patients, a total of 100 household members from 100 patients met the inclusion criteria. Overall, 80% of patients and 81% of household members had resided in the same area for more than ten years prior to the study. All household members had cohabited with the patients for more than five years, with 76% for more than ten years. Among patients, 53% were female and 47% male, while household members were evenly distributed (50% each gender). Patients had a mean age of 63.8 years (median = 66.0, SD = 12.7, range = 32–86) and household members had a mean age of 63.5 years (median = 66.0, SD = 13.7, range = 30–86).

Of all household members, 12% underwent serological testing without abdominal imaging. Reasons included patient refusal and incomplete external imaging. Eleven household members presented with valid recent findings from abdominal US, CT, or MRI scans, which did not reveal any suspicious findings. At our center, we performed abdominal US in 77 household members. In 75 of these, no lesion suspicious for AE could be found. After excluding the 12 participants who underwent serological testing without imaging, 88 participants had abdominal imaging data available (77 performed at our center plus 11 valid external findings). Of these 88 participants, 86 (98%) showed no lesion suggesting AE. In one participant, bowel gas could not be clearly distinguished from a hepatic lesion, prompting referral for a CT scan, which revealed no hepatic lesion. In another participant, a lesion could not be classified during the screening US due to overlying bowel gas. Therefore, MRI was performed, which demonstrated a benign cyst and ruled out AE.

In two participants, conventional US could not exclude an AE lesion. One lesion was considered a calcified, so-called aborted or died-out AE lesion. The other lesion was followed up with CEUS, which identified a hemangioma and excluded AE. Both participants were seronegative.

In total, US screening identified one possible case of AE, presenting as a calcified and therefore inactive lesion. Follow-up examination after 18 months showed that the lesion remained unchanged and that serology was persistently negative.

Regarding serology, six participants showed positivity or borderline results in one of the two serological screening tests. Four household members showed borderline or positive results for the *Echinococcus* IgG EIA screening test, and two for the more specific *Echinococcus* IgG Em2-Em18 test. None of the participants showed positive results in both initial serological tests. For these six participants, an *Echinococcus* western blot was performed, which showed negative results for all patients. Therefore, based on the serological screening, no patient was classified as possible AE, since this requires a positive serology in two different serological tests.

**Fig. 1:**
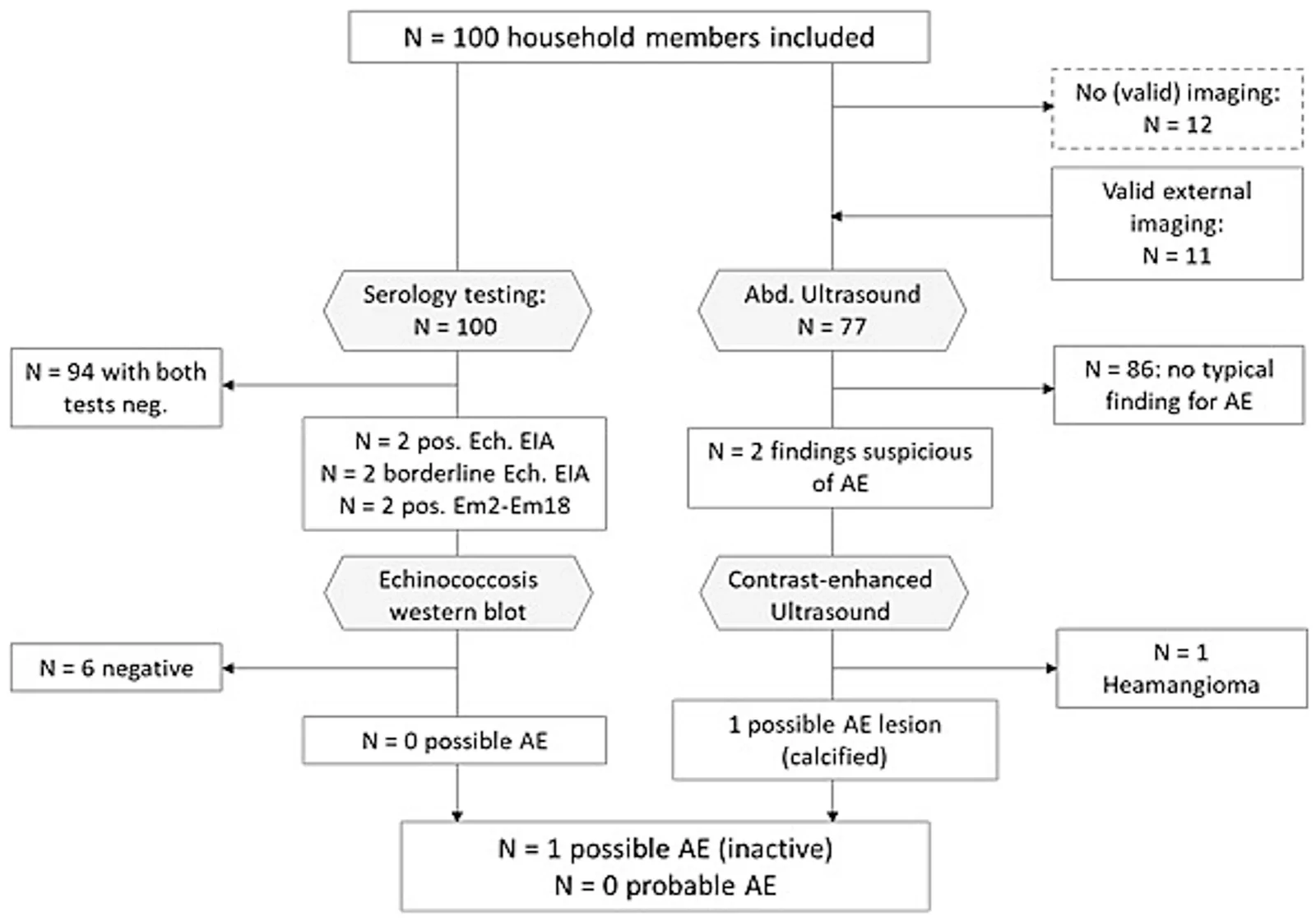
Flow chart with US and serological results.

The exact serological results are shown in Table 1. A flow chart reflecting the results is presented in Fig. 1.

**Table 1:** Positive serological results and respective US findings.

Participant number	*Echinococcus* IgG EIA⁎	*Echinococcus* IgG Em2-Em18⁎⁎	*Echinococcus* western blot	Abdominal ultrasound
1	11.7	Negative	Negative	No AE lesion
2	10.7	Negative	Negative	No AE lesion
3	16.5	Negative	Negative	No AE lesion
4	16.5	Negative	Negative	No AE lesion
5	Negative	2.0	Negative	No AE lesion
6	Negative	1.3	Negative	No AE lesion

⁎ Values <10 are considered negative, values from 11 to 15 borderline and values >15 positive.

⁎⁎ Values <1 are considered negative, values ≥1 positive.

## 4 Discussion

### 4.1 Principal findings

Our screening of household members who had lived with AE patients for at least five years prior to diagnosis identified one possible case of AE, consisting of a calcified and presumably inactive lesion. Follow-up after 18 months showed that the lesion remained stable and that serology was persistently negative, supporting the presumed inactivity of the lesion [17], [18].

Six patients showed positive or borderline serological results: four had positive or borderline *Echinococcus* IgG EIA results, and two had positive Em2-Em18 tests. Importantly, no patient was positive in both tests or in a subsequent western blot analysis. Therefore, none of these patients qualified as possible AE cases, and none had hepatic lesions suspicious of AE [19].

Serological findings require careful interpretation. A positive *Echinococcus* IgG EIA without Em2-Em18 positivity or hepatic lesions might represent nonspecific reactivity and does not necessarily indicate prior EM exposure. In household members with positive Em2-Em18 results, prior infection without clinical manifestation is possible, though nonspecific reactions are not excluded given the negative screening test and western blot in these cases.

### 4.2 Comparison with population-based screening studies

To contextualize our findings, we compared our results with epidemiological studies and screenings conducted in highly endemic areas:

During the last decades, the prevalence and incidence of AE have been rising [20]. The annual incidence rate for Germany is estimated at approximately 0.05, comparing to 0.06 in France, 0.08 in Austria, and 1.37 in Switzerland [21]. German prevalence rose from 0.05 per 100,000 (1992–1996) to 0.22 per 100,000 (2012–2016) [22], with southern regions showing the highest prevalence of 1.5–2.4 per 100,000 [23], [24] and up to 29.8 per 100,000 in certain hot spots [25]. The incidence in Germany increased from 0.12 per 100.000 (2004–2011) to 0.20 (2012–2019) with a rising number of urban cases [26]. A similar increase was observed in the high endemic region of Eastern France, with an estimated prevalence between 1972 and 1982 at approximately 2.5 per 100,000 inhabitants, rising to 6.6 per 100,000 between 1983 and 1993 [6]. This rise reflects both increased incidental detection through widespread imaging use and genuinely increased transmission at the human–animal–environment interface, driven by changes in human land use and expanding fox populations in urban and peri-urban ecosystems with increasing environmental burden of EM eggs [27], [28].

Our study revealed one possible case among 88 unrelated household members who received an abdominal US, corresponding to a prevalence of 1136 per 100,000, which exceeds the prevalence in the general population in Germany and southern regions. However, comparability is limited due to the small sample size, where each case significantly impacts prevalence calculations. Additionally, retrospective epidemiological studies likely underestimate true prevalence by missing asymptomatic lesions, and case clustering in endemic regions creates uneven geographic distribution. Therefore, we compare our findings with results from four major screenings in the following section.

Bresson-Hadni and colleagues [19] screened 7884 people in a highly endemic region in France using a sensitive and a specific ELISA based on the antigen Emc derived from the EM metacestode grown intraperitoneally in voles, and on the antigen Em2. They reported an overall seroprevalence (≥1 positive serological test) of 1.9% with 97 cases positive in one test and 55 positive in both tests (0.7%). From the 140 seropositive cases that received imaging, 13 showed hepatic lesion suspicious of AE (prevalence: 165 per 100,000 inhabitants) of which eight were active (102 per 100,000) and five were inactive (64 per 100,000). In the remaining seropositive cases, no lesion was detected.

Similarly, Romig et al. [29] screened 2560 inhabitants of a rural community in a high-risk area, applying both sensitive and specific serological testing as well as abdominal US. Serological testing included two ELISAs based on crude antigens: Emc, equivalent to Bresson-Hadni et al. [6], and Emf, derived from vesicular fluid of the metacestode grown in vitro. In total, 50 participants were seropositive (2.0%), of which five showed positivity in both serological tests (0.2%). Imaging revealed three AE lesions (117 per 100,000): one active AE lesion (39 per 100,000), and two inactive lesions (78 per 100,000). The remaining seropositive participants showed no suspicious hepatic lesion. In 36 of these seropositive cases, follow-up abdominal US after 30 months still revealed no evidence of AE [30].

Gottstein et al. [31] screened 2943 blood donors from 38 villages in Switzerland using a sensitive (*E. granulosus* hydatid fluid antigen) and a specific (Em2 ELISA) serological test. Results showed 39 seropositive cases (1.3%), of which 33 had a positive screening and six a positive specific test. No case was positive in both tests. Of all 39 patients with positive test, only 13 underwent imaging: of those with positive specific test, four received an abdominal US, revealing no lesions in three participants and a calcified inactive lesion in one person (34 per 100,000). Of the 33 participants with a positive screening test, only ten received an abdominal US, revealing cystic echinococcosis in one participant and no suspicious lesion in the remaining participants.

In 2002, Hänle et al. [32] conducted a screening study in a rural town in southwestern Germany using abdominal US and serological testing with Emc and Emf ELISAs. Among 2187 adults screened, no liver lesions suspicious for AE were detected. Serological testing was positive in 3.9% of participants, with 16 positive for Emc ELISA and 79 for Emf. Positive serum samples were later re-tested with the Em2-Em18 ELISA, which was negative in all cases. A follow-up examination after more than one year was performed in 44 seropositive individuals; none showed sonographic lesions suspicious for AE.

Pooling the French, Swiss and German data, 15,574 individuals were screened, yielding 288 seropositive cases (≥1 positive serological test), 9 active cases and 8 inactive cases of AE. This corresponds to an overall seroprevalence of 1.9% (95% CI 1.6–2.1%) and a prevalence of AE of 109 per 100,000 population (95% CI 64–174) in high-endemic areas, with 58 (95% CI 26–110) active and 51 (95% CI 22–101) inactive lesions per 100,000 population. The number needed to screen (NNS) to identify a case of AE in the general population in high-endemic areas is 916 (95% CI 572–1572). Specifically, NNS is 1730 (95% CI 912–3784) in the total population or 32 (95% CI 17–70) among seropositive individuals to identify one active case, and 1947 (95% CI 988–4509) in the total population or 36 (95% CI 19–83) among seropositive individuals to identify one inactive case. However, since only seropositive cases received imaging in the French and Swiss studies, the number of inactive cases is likely to be underestimated, as serology can be negative in these cases.

A summary of the data is presented in Table 2.

**Table 2:** Overview of seroprevalence and prevalence of AE in European screenings.

Study	Individuals Screened	Seroprevalence (positive in ≥1 test)	AE Lesions Detected	Prevalence of AE per 100,000	Prevalence of Active AE per 100,000
Bresson-Hadni et al. 1994, France	7884	1.9%	13 (8 active, 5 inactive)	165	102
Romig et al. 1999, Germany	2560	2.0%	3 (1 active, 2 inactive)	117	39
Gottstein et al. 2001, Switzerland	2943	1.3%	1 (inactive)	34	0
Hänle et al. 2009, Germany	2187	3.9%	0	0	0
**Pooled data from population-based screenings in Europe**	**∑ 15574**	**1.9% (1.6–2.1)**⁎	**∑ 17 (9 active, 8 inactive)**	**109 (64–174)**⁎	**58 (26–110)**⁎
This study (household members, Germany)	100	6.0%	1 (inactive)	1136	0

⁎ (95% CI).

The prevalence estimated in these screenings is considerably higher than that derived from retrospective epidemiological studies, which are prone to underestimating the occurence of AE, particularly in early or inactive stages that may remain undiagnosed in routine clinical care. Moreover, as noted above, the distribution of AE is highly heterogeneous. Nationwide incidence or prevalence figures therefore obscure substantial regional differences and may underestimate the burden in specific high-endemic areas.

Our study revealed six cases with single-test seropositivity without lesions suspicious of AE, corresponding to a higher overall seroprevalence (≥1 positive test) (6.0%) than earlier population screenings in high-endemic areas (1.9%). However, comparability might be limited by differences in the serological methods used. In our study, no participants were positive in both tests, similar to the Swiss study [31] but in contrast to the French and German screenings [19], [29]. We identified one possible AE lesion (1136 per 100,000), exceeding the prevalence reported in population-based screenings (109 per 100,000). However, this lesion was calcified and inactive, and no active lesions were found. This absence of active disease aligns with the Swiss findings [31] and the screening conducted by Hänle et al. [32] but differs from the screening performed by Romig et al. [29] and the French study [6], which detected 39–102 active cases per 100,000. As noted, our small sample size limits direct comparability with population-based screenings due to the substantial impact of individual cases on prevalence calculations.

### 4.3 Strenghts and limitations

This study represents, to our knowledge, the first systematic screening for AE among household members of patients with AE. Despite the rarity of the disease, we were able to invite a comparatively large cohort of eligible household contacts, and participation among those meeting the inclusion criteria was high. Nevertheless, the overall sample size remains limited for robust comparisons with population-based screening studies and for detailed subgroup analyses. In addition, we did not include biologically related household contacts, precluding assessment of potential genetic susceptibility; this should be addressed in future research.

## 5 Conclusion

Based on the higher seroprevalence (≥1 positive test) among household contacts compared to the general population in high-endemic areas, an increased risk of EM infection in this population cannot be excluded, as household members share the same human–animal–environment interface as their partners with AE and may therefore have similar exposure to EM eggs. Nevertheless, no active and only one inactive case was identified in our screening. Thus, even if the overall (sero)prevalence is elevated in this risk group, AE remains a rare disease. Although exposure to EM eggs is necessary for infection, genetic and immunological susceptibility are likely to influence whether infection becomes established and progresses to clinically detectable disease. AE is therefore best understood within a multifactorial model including both environmental exposure and host-related factors.

Our findings suggests that routine screening among household members cannot be recommended at present. If screening is requested by a patient's household member, abdominal US should be preferred over serological screening due to high false-positive rates seen in our and previous studies. Moreover, disease development in seropositive cases has not been demonstrated in two follow-up screenings conducted in Germany [30], [32]. However, serological confirmation remains necessary to confirm or exclude AE in suspicious imaging findings.

Nevertheless, as our anecdotal reports suggest, patients and clinicians should maintain awareness of a certain risk of AE in household members and pursue further diagnostics if symptoms or abnormal laboratory test results should occur.

## Use of artificial intelligence tools

The generative AI Claude Sonnet 4 was used to improve the language and style of several sentences or paragraphs in the abstract, the introduction and the discussion. The prompts used were ‘please check for language and grammar mistakes’ and ‘please improve the language flow of the following sentence(s)’.

## CRediT authorship contribution statement

**Lynn Peters:** Writing – original draft, Visualization, Project administration, Methodology, Investigation, Formal analysis, Data curation, Conceptualization. **Alexander Grunenberg:** Writing – review & editing, Validation, Investigation. **Giuseppe Zenatti:** Writing – review & editing, Validation, Investigation. **Wanjie Jiang:** Writing – review & editing, Validation, Project administration, Data curation. **Yasemin Özkan:** Writing – review & editing, Validation, Project administration, Data curation. **Nele Wellinghausen:** Writing – review & editing, Validation, Methodology, Investigation. **Teresa Rangel-Vivar:** Writing – review & editing, Validation, Investigation. **Andreas Binzberger:** Writing – review & editing, Validation, Investigation. **Wolfgang Kratzer:** Writing – review & editing, Validation, Investigation. **Peter Kern:** Writing – review & editing, Validation, Funding acquisition. **Benedikt Haggenmüller:** Writing – review & editing, Validation, Resources, Methodology, Investigation. **Beate Grüner:** Writing – review & editing, Validation, Supervision, Project administration, Methodology, Investigation, Conceptualization.

## Ethics declaration

Written informed consent to take part in the study and to publish the article has been obtained from all participants or their legal representatives. The privacy rights of participants have been observed.

This study included organ or tissue donors.

This study includes human biological material and consent was obtained by donors, or their next of kin or legal representatives, for use in this study and for publication of the article. The samples used in this research were not sourced from executed prisoners or prisoners of conscience.

This study was performed in compliance with relevant laws, regulatory frameworks and guidelines where the research took place.

This study was approved by the Ethikkommission der Universität Ulm.

(Approval No. 20/24)

## Funding statement

The study was partly funded by the 10.13039/501100001659Deutsche Forschungsgemeinschaft (DFG), grant number Ke 282/12 (to Prof. Peter Kern). The DFG was not involved in the study design, data collection, interpretation of data, writing of the report or the decision to submit the paper for publication.

## Declaration of competing interest

The authors declare the following financial interests/personal relationships which may be considered as potential competing interests: Prof. Dr. Peter Kern reports financial support was provided by 10.13039/501100001659Deutsche Forschungsgemeinschaft (DFG). If there are other authors, they declare that they have no known competing financial interests or personal relationships that could have appeared to influence the work reported in this paper.

## Data Availability

Data will be made available on request.
